# Supplementary material for: Molecular Liquids versus Ionic Liquids: The Interplay between Inter-Molecular and Intra-Molecular Hydrogen Bonding as Seen by Vaporisation Thermodynamics
Source: Molecules. 2023 Jan 5;28(2):539. doi: 10.3390/molecules28020539 (PMC9862684; doi:10.3390/molecules28020539)
Supplement: Supplementary file 1 [file molecules-28-00539-s001.zip › molecules-2102215-supplementary.pdf]

All samples used in this study were of commercial origin with purities of 98-99% as stated by the manufacturer. Only the samples of [N-C_2OH-Py][NTf_2], [N-C_3OH-Py][NTf_2], [N-C_4OH-Py][NTf_2], [N-C_2OH-Py][OMs] and [N-C_3OH-Py][OMs] were prepared and purified as described in our previous work [1]

Transpiration method

Absolute vapour pressures of piperidine-alcohols were measured using the transpiration method [2,3]. The main idea of this method is to saturate the gas stream flowing over the sample and to determine the amount of compound transferred by the gas within a given time. Approximately 0.5 to 0.8 g of the sample is mixed with glass beads (diameter 1 mm) and placed in the thermostatted U-shaped saturator. The glass beads are needed to enlarge the contact area between gas and sample. A stream of nitrogen at a well-defined flow rate was passed through the saturator at constant temperature (± 0.1 K), and the transported material was collected in a cold trap. The amount of the condensed substance was determined by GC. The saturation vapour pressure p at each temperature T_i was calculated from the amount of condensate collected within a definite period of time:

$$p = m_i \cdot R \cdot T_a / V \cdot M_i \;; \qquad V = (n_{N2} + n_i) \cdot R \cdot T_a / P_a \tag{S1}$$

where V is the volume of the gas phase consisting of the n_{N2} moles of the carrier gas and n_i mole of gaseous compound under study (with the molar mass M_i) at the atmospheric pressure P_a and the ambient temperature T_a. The volume of the carrier gas V_{N2} was determined by the digital flow rate sensor from integration with a microcontroller. We used the Honeywell S&C - HAFBLF0200C2AX5 digital flow rate sensor with uncertainty at the level of 2.5 %. The flow rate of the nitrogen stream was also controlled by using a soap bubble flow meter (HP soap film flowmeter (model 0101-0113)) and optimized in order to reach the saturation equilibrium of the transporting gas at each temperature under study. The volume of the carrier gas V_{N2} was readied from the digital flow rate sensor. The amount of the compound under investigation n in the carrier gas was estimated at each temperature using the ideal gas law.

Before starting the vapour pressure measurements, the sample was first pre-conditioned at 310-320 K (within about one hour) in order to remove possible traces of water. The saturator was then kept at 310-315 K (to remove possible traces of volatile compounds). In order to assure the competition of pre-conditioning at the selected temperature, three samples were taken during the sample flashing at and analysed by the GC. A constant vapour pressure at this temperature indicated that the transpiration experiments could begin. GC analysis of the transported material did not reveal any additional contamination. The absence of impurities and decomposition products was re-checked by GC analysis of the saturator content at the end of the entire series of experiments.

Thermodynamics of vaporisation/sublimation

Experimental vapour pressures measured in this work (as well as those from the literature) have been used to obtain the enthalpies of vaporisation using the following equation:

$$\Delta_l^g H_m^o(T) = -b + \Delta_l^g C_{p,m}^o \times T \tag{S2}$$

Experimental vapour pressures temperature dependences were also used to derive the vaporisation entropies at temperatures T by using the following equation:

$$\Delta_l^g S_m^o(T) = \Delta_l^g H_m^o / T + R \times \ln(p_i / p^o) \tag{S3}$$

with p^o = 0.1 MPa. Coefficients a and b of Equation (1), $\Delta_l^g H_m^o(T)$ and $\Delta_l^g S_m^o(T)$ values are collected in Table 1. The Equations S2-S3 are also valid for the treatment of vapour pressures measured over the solid sample, giving the standard molar enthalpy of sublimation $\Delta_{cr}^g H_m^o(T)$ and the standard molar sublimation entropy $\Delta_{cr}^g S_m^o(T)$. In this case in Equations S2-S3 we used the value $\Delta_{cr}^g C_{p,m}^o$ instead of the $\Delta_l^g C_{p,m}^o$-values (see Table S2). The combined uncertainties of the vaporisation enthalpies include uncertainties from the experimental conditions of transpiration. uncertainties in vapour pressure and

uncertainties due to the temperature adjustment to T = 298.15 K as described elsewhere [4,5]. The compilation of the standard molar enthalpies of vaporisation at the reference temperature T = 298.15 K, calculated according to Equation (S2) is given in Table 1.

Temperature adjustments of sublimation/vaporisation enthalpies.

According to general practice, all thermochemical quantities must be represented at the reference temperature T = 298.15 K. Sublimation/vaporisation enthalpy derived from vapour pressure measurements are usually referenced to the average temperature, T_{av}, of the experimental range. In any case, they need to be adjusted to T = 298.15 K using a Kirchhoff´s Law. The isobaric heat capacity differences $\Delta_{cr,l}^{g}C_{p,m}^{o}$ are required for temperature adjustment of vaporisation/sublimation enthalpies according to Kirchhoff's rule. The simple and straightforward method was developed by Chickos and Acree [6]. They suggested an empirical way to assess $\Delta_{l}^{g}C_{p,m}^{o}$–values by equation:

$$\Delta_{l}^{g}C_{p,m}^{o}(298.15\ \mathrm{K}) = -0.26 \times C_{p,m}^{o}(\mathrm{liq}, 298.15) - 10.58 \quad \text{(S4)}$$

where $C_{p,m}^{o}$(liq, 298.15 K)-data are of experimental origin or they can be also estimated according to the group-additivity procedure [6].

$$\Delta_{cr}^{g}C_{p,m}^{o}(298.15\ \mathrm{K}) = -0.15 \times C_{p,m}^{o}(\mathrm{cr}, 298.15) - 0.72 \quad \text{(S5)}$$

where $C_{p,m}^{o}$(cr, 298.15 K)-data are of experimental origin or they can be also estimated according to the group-additivity procedure [7].

Table S1

Results of the transpiration method: absolute vapor pressures, p, standard molar vaporisation enthalpies, $\Delta_{l}^{g}H_{m}^{o}$, and standard molar vaporisation entropies, $\Delta_{l}^{g}S_{m}^{o}$

T/ K[a]	m/ mg[b]	$V(N_2)$[c] / dm^3	T_a/ K[d]	Flow/ dm^3·h^{-1}	p / Pa[e]	$u(p)$/ Pa[f]	$\Delta_{l}^{g}H_{m}^{o}(T)$/ kJ·mol^{-1}	$\Delta_{l}^{g}S_{m}^{o}(T)$/ J·K^{-1}·mol^{-1}
1-(2-hydroxyethyl)-piperidine: $\Delta_{l}^{g}H_{m}^{o}$(298.15 K) = (58.1±0.4) kJ·mol^{-1}								
$\ln(p/p_{ref}) = \frac{299.0}{R} - \frac{80122.4}{RT} - \frac{73.6}{R}\ln\frac{T}{298.15}$; p_{ref} = 1 Pa								
283.2	2.75	4.724	287.2	3.88	10.76	0.29	59.3	133.4
285.3	2.47	3.535	288.2	3.60	12.98	0.35	59.1	132.9
286.2	2.83	3.689	287.2	3.88	14.18	0.38	59.1	132.7
288.4	2.57	2.816	288.2	3.60	16.93	0.45	58.9	132.1
290.3	2.95	2.714	287.7	2.86	20.15	0.53	58.8	131.7
292.2	2.68	2.097	287.7	3.60	23.72	0.62	58.6	131.3
294.2	2.92	1.991	288.2	3.32	27.27	0.71	58.5	130.5
295.2	2.86	1.770	293.2	2.80	30.56	0.79	58.4	130.6
296.4	2.76	1.539	286.7	3.08	33.19	0.85	58.3	130.2
297.2	4.71	2.473	288.2	3.62	35.32	0.91	58.3	129.9
298.3	2.92	1.417	288.2	5.00	38.27	0.98	58.2	129.6
298.3	2.94	1.409	292.2	2.73	39.32	1.01	58.2	129.8
299.4	2.77	1.258	293.2	2.80	41.58	1.06	58.1	129.3
300.4	4.96	2.039	286.7	2.72	44.94	1.15	58.0	129.1
301.3	2.67	1.026	286.7	3.08	48.09	1.23	58.0	128.8
303.2	2.70	0.932	292.7	2.80	54.53	1.39	57.8	128.2
303.3	2.78	0.909	292.2	2.73	57.61	1.47	57.8	128.6
305.3	2.83	0.821	293.2	3.08	65.08	1.65	57.7	127.9
306.2	2.92	0.788	296.7	2.70	70.78	1.79	57.6	127.8
307.4	2.56	0.652	296.7	1.96	74.84	1.90	57.5	127.2
308.2	2.97	0.687	292.2	2.75	81.38	2.06	57.4	127.3
310.2	2.06	0.421	293.2	1.69	92.50	2.34	57.3	126.7
310.3	2.68	0.546	297.2	1.99	93.97	2.37	57.3	126.7
311.4	2.56	0.489	297.2	1.96	100.07	2.53	57.2	126.3

312.3	2.42	0.421	293.2	1.69	108.23	2.73	57.1	126.2
313.3	2.89	0.477	297.2	1.69	115.76	2.92	57.1	126.0
313.3	3.77	0.618	296.7	2.75	116.34	2.93	57.1	126.0
316.3	5.27	0.694	295.7	2.69	144.58	3.64	56.8	125.4
318.2	5.16	0.590	296.7	1.61	166.79	4.19	56.7	125.1
320.3	5.48	0.550	295.7	1.61	189.58	4.76	56.6	124.5
323.2	5.13	0.429	296.7	1.61	228.07	5.73	56.3	123.8
1-(3-hydroxypropyl)-piperidine: $\Delta_l^g H_m^o$(298.15 K) = (62.1±0.4) kJ·mol^{-1}								
$\ln(p/p_{ref}) = \frac{312.1}{R} - \frac{86476.3}{RT} - \frac{81.9}{R}\ln\frac{T}{298.15}$; p_{ref} = 1 Pa								
283.4	2.62	11.91	296.8	6.20	3.84	0.10	63.3	138.7
283.5	2.62	11.76	297.0	5.85	3.89	0.10	63.3	138.7
285.3	2.89	10.79	292.5	4.53	4.60	0.12	63.1	138.1
288.3	2.76	7.898	296.4	5.85	6.07	0.18	62.9	137.4
291.3	2.88	6.438	295.8	5.52	7.74	0.22	62.6	136.3
293.3	2.80	5.265	295.2	5.85	9.16	0.25	62.5	135.7
298.2	3.17	3.900	295.3	5.85	14.00	0.37	62.1	134.3
303.1	3.14	2.535	295.0	5.85	21.26	0.56	61.7	133.1
308.1	2.89	1.573	295.0	4.29	31.55	0.81	61.2	131.8
313.0	2.80	1.073	294.5	2.22	44.68	1.14	60.8	130.2
317.9	2.82	0.740	294.4	2.22	65.01	1.65	60.4	129.1
320.9	2.79	0.586	295.6	1.38	81.70	2.07	60.2	128.4
322.9	3.23	0.592	293.9	2.22	92.88	2.35	60.0	127.9
324.9	2.87	0.459	295.5	1.38	107.04	2.70	59.9	127.4
328.9	2.84	0.335	293.1	1.00	144.17	3.63	59.5	126.7
331.8	2.87	0.284	292.0	1.00	170.99	4.30	59.3	125.7
334.8	3.20	0.259	291.7	1.00	208.55	5.24	59.1	125.1
337.8	3.75	0.255	292.2	1.00	248.98	6.25	58.8	124.3

[a] Saturation temperature measured with the standard uncertainty ($u(T)$ = 0.1 K).
[b] Mass of transferred sample condensed at T = 273 K.
[c] Volume of nitrogen ($u(V)$ = 0.005 dm^3) used to transfer m ($u(m)$ = 0.0001 g) of the sample. Uncertainties are given as standard uncertainties.
[d] T_a is the temperature of the soap bubble meter used for measurement of the gas flow.
[e] Vapour pressure at temperature T, calculated from the m and the residual vapour pressure at the condensation temperature calculated by an iteration procedure.
[f] Standard uncertainties were calculated with $u(p/\text{Pa})$ = 0.005 +0.025(p/Pa) for pressures below 5 Pa and with $u(p/\text{Pa})$ = 0.025 + 0.025(p/Pa) for pressures from 5 to 3000 Pa. The standard uncertainties for T, V, p, m, are standard uncertainties with 0.683 confidence level. Uncertainty of the vaporisation enthalpy $U(\Delta_l^g H_m^o)$ is the expanded uncertainty (0.95 level of confidence) calculated according to procedure described elsewhere [4,5]. Uncertainties include uncertainties from the experimental conditions and the fitting equation, vapour pressures, and uncertainties from adjustment of vaporisation enthalpies to the reference temperature T = 298.15 K.

Table S2

Vapor pressures, p, at different temperatures compiled from the literature [8], standard molar vaporisation enthalpies, $\Delta_l^g H_m^o$, and standard molar vaporisation entropies, $\Delta_l^g S_m^o$

T/ K[a]	p/ Pa[e]	$\Delta_l^g H_m^o(T)$/ [a] kJ·mol^{-1}	$\Delta_l^g S_m^o(T)$/ J·K^{-1}·mol^{-1}
1-(2-hydroxyethyl)-piperidine: $\Delta_l^g H_m^o$(298.15 K) = (56.4±1.7) kJ·mol^{-1}			
$\ln(p/p_{ref}) = \frac{294.7}{R} - \frac{78354.5}{RT} - \frac{73.6}{R}\ln\frac{T}{298.15}$; p_{ref} = 1 Pa			
331	533	54.0	119.5
338	667	53.5	116.5
340	667	53.3	115.1
353	1600	52.4	113.9

355	1600	52.2	112.6
362	2666	51.7	112.6
362	2666	51.7	112.6
363	2133	51.6	110.2
363	2133	51.6	110.2
363	2266	51.6	110.7
363	2266	51.6	110.7
364	2666	51.6	111.4
364	2666	51.6	111.4
365	2133	51.5	109.0
365	2133	51.5	109.0
365	2266	51.5	109.5
365	2266	51.5	109.5
368	2666	51.3	109.1
368	2666	51.3	109.1
369	2666	51.2	108.5
472	101325	43.6	92.5
472	97992	43.6	92.2
475	101325	43.4	91.4
477	101325	43.2	90.7
481	101325	42.9	89.4

1-(3-hydroxypropyl)-piperidine: $\Delta_{l}^{g}H_{m}^{o}$(298.15 K) = (62.3±2.3) kJ·mol^{-1}

$\ln{(p/p_{ref})} = \frac{311.4}{R} - \frac{86745.6}{RT} - \frac{81.9}{R}\ln\frac{T}{298.15}$; p_{ref} = 1 Pa

362	800	57.1	117.5
369	1467	56.5	118.0
371	1467	56.3	116.7
373	1600	56.2	116.2
375	1600	56.0	114.9
378	2133	55.8	115.5
378	1600	55.8	113.1
380	2133	55.6	114.3
382	2533	55.4	114.5
383	2533	55.4	113.9
387	2533	55.0	111.6
388	2666	55.0	111.4
390	3333	54.8	112.2
395	3333	54.4	109.3
397	4000	54.2	109.8
400	4000	54.0	108.1
422	9066	52.2	103.6
422	9066	52.2	103.6
496	99992	46.1	92.9
496	101325	46.1	93.0
498	99992	45.9	92.2
501	99992	45.7	91.2

1-(4-hydroxybutyl)-piperidine: $\Delta_{l}^{g}H_{m}^{o}$(298.15 K) = (70.0±2.6) kJ·mol^{-1}

$\ln{(p/p_{ref})} = \frac{332.4}{R} - \frac{96887.4}{RT} - \frac{90.2}{R}\ln\frac{T}{298.15}$; p_{ref} = 1 Pa

348	133	65.5	133.0
393	1333	61.4	120.3
400	2400	60.8	120.9

402	2400	60.6	119.7
402	2400	60.6	119.7
406	2666	60.3	118.2
407	2666	60.2	117.6
518	101325	50.2	97.0

3-phenyl-1-propanol: $\Delta_{\mathrm{l}}^{\mathrm{g}}H_{\mathrm{m}}^{\mathrm{o}}$(298.15 K) = (75.1±2.4) kJ·mol^{-1}

$\ln(p/p_{ref}) = \frac{337.8}{R} - \frac{100010.7}{RT} - \frac{83.6}{R}\ln\frac{T}{298.15}$; p_{ref} = 1 Pa

338	40	71.8	147.2
339	40	71.7	146.4
371	267	69.0	136.7
373	267	68.8	135.2
391	1600	67.3	137.8
392	1600	67.2	137.1
392	1600	67.2	137.1
394	1600	67.1	135.9
395	2000	67.0	137.1
395	1867	67.0	136.5
396	1733	66.9	135.2
396	1733	66.9	135.2
398	2666	66.7	137.5
398	2133	66.7	135.7
408	3600	65.9	133.9
408	2666	65.9	131.4
409	2533	65.8	130.4
410	2533	65.7	129.8
413	4266	65.5	132.3
413	3600	65.5	130.9
503	101325	58.0	115.3
503	101325	58.0	115.3
504	101325	57.9	114.9
506	98658	57.7	113.9
506	101325	57.7	114.2
508	101325	57.5	113.4
508	98658	57.5	113.2
509	101325	57.5	113.0
510	101325	57.4	112.6
513	101325	57.1	111.5

4-phenyl-1-butanol: $\Delta_{\mathrm{l}}^{\mathrm{g}}H_{\mathrm{m}}^{\mathrm{o}}$(298.15 K) = (80.2±3.0) kJ·mol^{-1}

$\ln(p/p_{ref}) = \frac{335.2}{R} - \frac{107785.1}{RT} - \frac{92.5}{R}\ln\frac{T}{298.15}$; p_{ref} = 1 Pa

367	133	73.8	146.1
368	133	73.7	145.4
373	267	73.3	147.2
390	800	71.7	143.7
396	1133	71.2	142.4
397	1200	71.1	142.2
398	1067	71.0	140.6
399	1067	70.9	139.9
400	1333	70.8	141.1

400	1200	70.8	140.2
407	1600	70.1	137.9
408	1867	70.0	138.6
408	1600	70.0	137.3
409	1733	70.0	137.3
410	1733	69.9	136.7
413	1867	69.6	135.4
413	1867	69.6	135.4
515	101325	60.1	116.9
521	101991	59.6	114.5

[a] Uncertainties of the vaporisation enthalpies are expressed as the standard uncertainties. They include uncertainties from the fitting equation, and uncertainties from temperature adjustment to T = 298.15 K. Uncertainties in the temperature adjustment of vaporisation enthalpies to the reference temperature T= 298.15 K are estimated to account with 20 % to the total adjustment.

Table S3

Compilation of data on molar heat capacities $C^{o}_{p,m}$(liq) and heat capacity differences $\Delta^{g}_{l}C^{o}_{p,m}$ at T = 298.15 K (in J·K^{-1}·mol^{-1})

ILs	$C^{o}_{p,m}$(liq)	Equation for $\Delta^{g}_{l}C^{o}_{p,m}$	$\Delta^{g}_{l}C^{o}_{p,m}$
		**Molecular liquids**	
1-(2-hydroxyethyl)-piperidine	242.4 [a]	$\Delta^{g}_{l}C^{o}_{p,m}$ = -0.26×$C^{o}_{p,m}$(liq) -10.58	-73.6 [b]
1-(3-hydroxypropyl)-piperidine	274.3 [a]		-81.9 [b]
1-(4-hydroxybutyl)-piperidine	306.2 [a]		-90.2 [b]
3-phenyl-1-propanol	280.7 [9]		-83.6 [b]
4-phenyl-1-butanol	314.5 [9]		-92.5 [b]
		**Ionic liquids**	
[N-C_2-Py][NTf_2]	508 [10]		-61 [10]
[N-C_3-Py][NTf_2]	540[10]		-66 [10]
[N-C_4-Py][NTf_2]	572 [11]		-70 [10]
[N-C_5-Py][NTf_2]	604[10]		-73 [10]
[N-C_6-Py][NTf_2]	636[10]		-77 [10]

[N-C$_2$OH-Py][NTf_2]	564 [c]	$\Delta_l^g C_{p,m}^o$ = -0.1219×$C_{p,m}^o$(liq) +0.3	-68 [d]
[N-C$_3$OH-Py][NTf_2]	596 [c]		-72 [d]
[N-C$_4$OH-Py][NTf_2]	628 [c]		-76 [d]
[N-C$_3$OH-Py][DCA]	378 [e]	$\Delta_l^g C_{p,m}^o$ = -0.0908×$C_{p,m}^o$(liq) – 33.5	-81 [f]
[N-C$_3$OH-Py][BF4]	366.5 [e]	$\Delta_l^g C_{p,m}^o$ = -0.0908×$C_{p,m}^o$(liq) – 33.5	-67 [f]
[N-C$_2$OH-Py][OMs]	354.4 [e]	$\Delta_l^g C_{p,m}^o$ = -0.0908×$C_{p,m}^o$(liq) – 33.5	-74 [f]
[N-C$_2$OH-Py][OMs]	385.8 [e]		-81 [f]

[a] Calculated by the group-contribution procedure developed by Chickos *et al.* [7]
[b] Calculated according to the procedure developed by Acree and Chickos [6].
[c] Calculated by the group-contribution procedure developed by Chickos *et al.* [7] and the reliable $C_{p,m}^o$(liq) for [N-C$_4$-Py][NTf_2] reported by Diedrichs and Gmehling [11]
[d] Calculated according to the equation given in column 3, developed from data for [N-C$_n$-Py][NTf_2] given in this table.
[e] Calculated according to the procedure developed by Ahmadi et al. [12].
[f] Calculated according to the equation given in column 3, developed from data for pyridinium based ionic liquids [13].

Table S4

Compilation of enthalpies of vaporisation; $\Delta_l^g H_m^o$, derived in this work and from the data available in the literature.

Compound	M[a]	*T*- range	$\Delta_l^g H_m^o(T_{av})$	$\Delta_l^g H_m^o$(298.15 K)[b]	Ref.
CAS		K	kJ·mol^{-1}	kJ·mol^{-1}	
1-(2-hydroxyethyl)-piperidine	C	326.8	68.8±0.4	(64.2±0.8)	[14]
3040-44-6	T	283.2-323.2	57.9±0.2	58.2±0.3	Table S1
	BP	331-481	48.4±0.7	56.4±1.7	Table S2
				**58.1±0.4** [c]	average
1-(3-hydroxypropyl)-piperidine	T	283.4-337.8	61.3±0.1	62.1±0.3	Table S1
104-58-5	BP	362-501	51.6±0.9	62.3±2.3	Table S2
				**62.1±0.4** [c]	average
1-(4-hydroxybutyl)-piperidine	BP	348-518	58.4±1.1	70.0±2.6	Table S2
4672-11-1					
3-phenyl-1-propanol (122-97-4)	BP	338-513	64.1±1.0	75.1±2.4	Table S2
4-phenyl-1-butanol (3360-41-6)	BP	367-521	67.2±1.4	80.2±3.0	Table S2

[a] Techniques: T = transpiration method; C = Calvet microcalorimetry; BP = derived from boiling temperatures at different pressures available in the literature [8].
[b] Vapor pressures available in the literature were treated using ?Equations (S2) and (S3) with help of heat capacity differences from Table S3 to evaluate the enthalpy of vaporisation at 298.15 K in the same way as our own results in Table S1. Uncertainties of the vaporisation enthalpies $u(\Delta_l^g H_m^o)$ are the standard uncertainty calculated according to procedure described elsewhere [4,5].
[c] Weighted mean value (uncertainties were taken as the weighing factor). Values in parenthesis were excluded from the calculation of the mean. Values in bold are recommended for further thermochemical calculations.

Table S5

Correlation of vaporisation enthalpies, $\Delta_l^g H_m^o$(298.15 K), of alkyl-piperidines with their Kovats indices (J_x)

Compound	J_x [a]	$\Delta_l^g H_m^o$(298 K)$_{exp}$ kJ·mol^{-1}	$\Delta_l^g H_m^o$(298 K)$_{calc}$ [b] kJ·mol^{-1}	Δ[c] kJ·mol^{-1}
N-methyl-piperidine	766	36.8±0.6 [15]	36.9	-0.1
N-ethyl-piperidine	850	40.8±0.6 [16]	40.9	-0.1
N-propyl-piperidine	930	44.9±0.4 [16]	44.6	0.3
N-butyl-piperidine	1025	48.9±0.2 [16]	49.1	-0.2
N-pentyl-piperidine	1122		**53.7**	
N-hexyl-piperidine	1220		**58.3**	

[a] Kovats indices, J_x, on the standard non-polar column OV-101 [17].
[b] Calculated using equation: $\Delta_l^g H_m^o$(298.15 K) /(kJ·mol^{-1}) = 0.0471×J_x + 0.8 with (R^2 = 0.9984) with the assessed uncertainty of ± 1.0 kJ·mol^{-1} (expanded uncertainty 0.95 level of confidence).
c $\Delta_l^g H_m^o$(298 K)$_{exp}$ − $\Delta_l^g H_m^o$(298 K)$_{calc}$

***Quartz Crystal Microbalance (QCM)*:**

Vapor pressures and standard molar enthalpies of vaporization were measured by using the QCM method [18]. A sample of an IL was placed in an open cavity (Langmuir evaporation) inside of the thermostat block and it was exposed to vacuum (10^{-5} Pa) with the whole open surface of the loaded compound. The QCM-sensor was mounted directly above the measuring cavity containing the sample. Along the vaporization into high vacuum, a certain amount of sample was condensed on the quartz crystal surface.

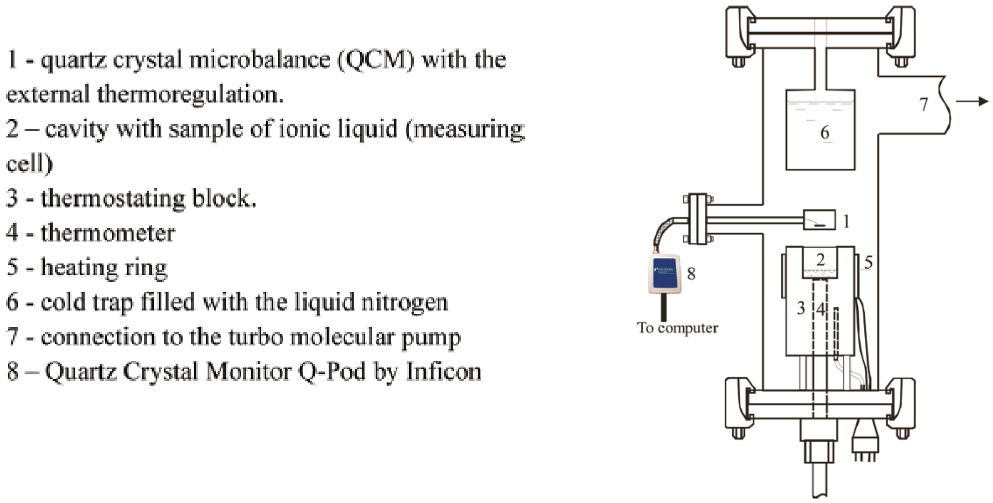

Figure S1. The scheme of the QCM experimental setup from [18].

The change of the vibrational frequency Δf was recorded. It is directly related to the mass deposition Δm on the QCM according to the Sauerbrey equation [19]:

$$\Delta f = -C \times f^2 \times \Delta m \times S_C^{-1} \qquad \text{(S6)}$$

where f is the fundamental frequency of the crystal (6 MHz in this case) with $\Delta f << f$, S_C is the surface of the crystal, and C is a constant [19]. The measured frequency change rates ($df/d\tau$) can be used for calculation of absolute vapor pressures p_s according to equation:

$$p_s = K' \frac{df}{dt}\sqrt{\frac{T}{M}}. \qquad \text{(S7)}$$

where the $K' = (9.5 \pm 1.1)\cdot 10^{-6}$ Pa·s·kg$^{1/2}$·Hz^{-1}·K$^{-1/2}$·mol$^{-1/2}$ [20] is the empirical calibration constant including all parameters involved in Equation 1, as well as the all apparatus geometry specific parameters. Calibration of the set up was performed with the help of reliable vapor pressure data on [C_nPy][NTf_2], [$C_nC_n$im][NTf_2], and [$C_n$mim][NTf_2] series of ionic liquids. Standard molar enthalpy of vaporization, $\Delta_l^g H_m^o(T_0)$, was calculated as follows:

$$\ln\left(\frac{df}{dt}\sqrt{T}\right) = A' - \frac{\Delta_l^g H_m^o(T_0) - \Delta_l^g C_{p_m}^{\ o} T_0}{R}\left(\frac{1}{T} - \frac{1}{T_0}\right) + \frac{\Delta_l^g C_{p_m}^{\ o}}{R}\ln\left(\frac{T}{T_0}\right) \qquad \text{(S8)}$$

where T_0 is an arbitrarily chosen reference temperature (T_0 average temperature of experimental range of the QCM study) and A' is the empirical constant. The value $\Delta_l^g C_{p,m}^o = C_{p,m}^o(g) - C_{p,m}^o(l)$ is the difference between the molar heat capacities of the gaseous $C_{p,m}^o(g)$ and the liquid phase $C_{p,m}^o(l)$ respectively. The vaporization enthalpy $\Delta_l^g H_m^o$ (T) at any temperature is calculated according to Kirchhoff´s equation:

$$\Delta_l^g H_m^o(T) = \Delta_l^g H_m^o(T_0) + \Delta_l^g C_{p,m}^{\circ}(T - T_0) \qquad \text{(S9)}$$

A typical experiment was performed in a few consequent series with increasing and decreasing temperature steps. Every series included from 7 to 11 temperature points of mass loss rate determination. Such a procedure allowed for detection of any possible decomposition during frequency loss rate ($df/d\tau$) measurements. Reproducibility of results was established in series of randomly performed experimental runs. The study was considered as completed when the $\Delta_l^g H_m^o(T)$-values derived from the temperature-dependent rates ($df/d\tau$) achieved the level of uncertainty of ±1 kJ·mol^{-1}. The absence of decomposition of IL under experimental conditions was controlled by using spectroscopy. The residual amount of IL in the cavity, as well as the IL-deposit on QCM were analyzed by ATR-IR spectroscopy.

TGA measurements of vaporization enthalpy.

We used a Perkin Elmer Pyris 6 TGA to determine $\Delta_l^g H_m^{\circ}$ from the temperature dependence of the mass loss rate measurements. About 50-70 mg of the IL sample was placed in a plain platinum crucible inside of the measuring head of the TGA. The sample was step-wise heated and a mass loss from the crucible was recorded at each isothermal step. Isothermal mass loss rate *dm*/*dt* was monitored in the temperature range 513-586 K at a nitrogen flow rate of 140 ml·min^{-1}. According to the results reported in our previous work [21], the optimal conditions for the reliable determination of vaporization enthalpies of ILs were as follows:

- mass loss *dm*/*dt* at each temperature - 0.1-0.8 mg;
- duration of isothermal steps - at least 10 min;
- temperature range - at least 60 K;

Prior to the measurement of vaporization enthalpy, a careful conditioning of the sample inside the TGA have been performed. A heating ramp of 10 K·min^{-1} was used, followed by a 4 h static hold period at 423 K, allowing for the slow removal of volatile impurities and traces of water prior to a stepwise isothermal runs. The conditioning was repeated until a reproducible mass loss within two consequent runs was recorded. An absence of decomposition of the IL in the experimental conditions was confirmed by ATR-IR spectroscopy. No changes in the spectra taken from the initial and the residual IL in the crucible were detected for the ILs under study. The detailed experimental procedure has been elaborated in our lab and it was reported elsewhere [21].

Gas-Chromatographic method (GC) [22]*:*

In this work we follow the Flory-Huggins theory, which is the main basis of solution and blend thermodynamics [23]. The Flory-Huggins equation handles molecules that are similar chemically, but differ greatly in size. A key value of this theory is a parameter χ_{12} quantifying the enthalpic interactions between the components *1* and *2*. The activity coefficient at infinite dilution γ_1^{∞} is linked to the Flory−Huggins interaction parameter χ_{12} (at infinite dilution) according to equation [24]:

$$\chi_{12} = \ln\left(\frac{273.15\,\gamma_1^{\infty} M_2}{T M_1}\right) - \left(1 - \frac{V_1^*}{V_2^*}\right) + \ln\left(\frac{\rho_1}{\rho_2}\right) \quad \text{(S10)},$$

where M_1 and M_2 are the molecular weight of solute and solvent, V_1* and V_2* and ρ_1 and ρ_2 are the molar volume and density of solute and solvent, respectively. The Flory–Huggins interaction parameters, χ_{12} is related to the Hildebrandt solubility parameters δ [24]:

$$\chi_{12} = \frac{V_1^*(\delta_1 - \delta_2)^2}{RT} \quad \text{(S11)},$$

where δ_2 is the solubility parameter of the IL (solvent) and δ_1 is the solubility parameter of the solute, *R* is the universal gas constant, *T* is the arbitrary temperature, V_1^* is the molar volume of the solute at the selected temperature. Solubility parameters are the numerical values that are responsible for the strength of the intermolecular interactions between solute and solvent molecules. The solubility parameters have been widely used because they help to assess the solvation powers of solvents.

The Hildebrand or total solubility parameter (δ_i) is defined as follows [25]:

$$\delta_i = [(\Delta_\mathrm{l}^\mathrm{g} H_\mathrm{m}^\mathrm{o} - RT)/V_\mathrm{m}]^{0.5} \quad \text{(S12)},$$

where V_m is the molar volume, $\Delta_\mathrm{l}^\mathrm{g} H_\mathrm{m}^\mathrm{o}$ is the standard molar enthalpy of vaporization, *R* is the ideal gas constant, and *T* is the temperature. Vaporization enthalpies, $\Delta_\mathrm{l}^\mathrm{g} H_\mathrm{m}^\mathrm{o}$, of ionic liquids required for calculations δ_2 at *T* = 298.15 K have been evaluated in Table 4, main text). Vaporization enthalpies, $\Delta_\mathrm{l}^\mathrm{g} H_\mathrm{m}^\mathrm{o}$, of molecular solutes required for calculations δ_1 at *T* = 298.15 K were taken from the literature [26]. Values of the solubility parameters δ_1 and δ_2 have been calculated according to Equation (S12) with help of experimental data on vaporization enthalpies for the solutes[26]. Density-values for the solutes were taken from the compilation by Lide [27] and for ILs from the original literature. Finally, the algebraic rearrangement of Equation (S11) gives

$$\frac{\delta_1^2}{RT} - \frac{\chi_{12}}{V_1^*} = \left(\frac{2\delta_2}{RT}\right)\delta_1 - \frac{\delta_2^2}{RT} \quad \text{(S13)}$$

As it was shown in a typical case (see Figure S2), when the left side of Equation (S13) is plotted against δ_1, then the mathematical term $2\delta_2/(RT)$ is the slope of the line and the module $-\delta_2^2/(RT)$ is its

intercept. Using linear regression of the experimental data, the slope or intercept can be used to determine δ_2.

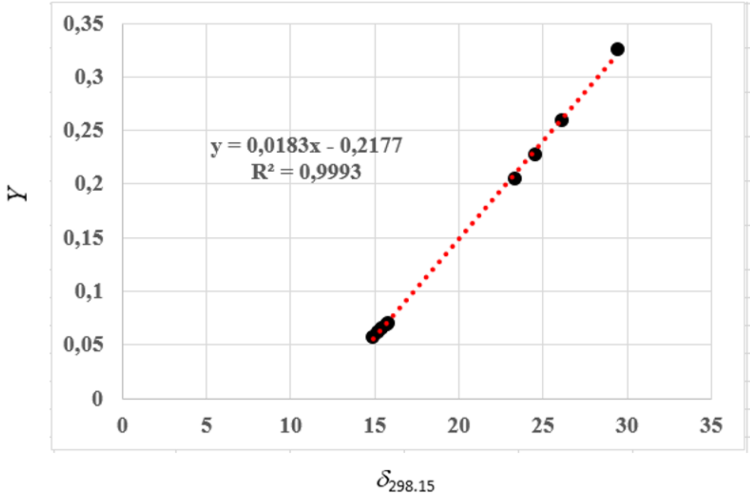

Figure S2 Regression of solubility parameters $\delta_{298.15}$ of different solutes and Y-module ($Y = (\delta_1)^2/(RT) - \chi_{12}/V_1^*$ is the left part of Equation (13)) for [N-C_3OH-Py][NTf_2] derived from experimental γ_1^∞-values [28].

We fitted Equation (13) with the solubility parameters δ_2 derived from primary γ_1^∞-values for the [C_nPy][Anion] available in the literature (the references for the primary γ_1^∞-values are given in Tables S6 and S7). It turned out, that for each data set, the solubility parameters δ_2 obtained using the slope and intercept were in agreement with each other within 3%. Thus, the δ_2-values can be estimated as the average from the slope and the intercept. Thus, the vaporization enthalpy, $\Delta_l^g H_m^o$(298.15 K), of an IL under study was calculated using the averaged δ_2-value as follows:

$$\Delta_l^g H_m^o(T) = [\delta_2^2 \times V_m + RT] \quad \text{(S14)},$$

where all values, including V_m are referenced to an arbitrary temperature T, which is 298.15 K in this work. And now these $\Delta_l^g H_m^o$(298.15 K)-results "indirectly" derived with help of the primary γ_1^∞-values can be used for comparison with the "direct" experimental results on vaporization enthalpies obtained by the conventional methods (see Tables S6 and S7).

Table S6

Data used for regression with Equation (S13) for [N-C_3OH-Py][NTf_2]: solubility parameters δ_1 (at 298.15 K) of different solutes and the left part of Equation (S13).

[N-C_3OH-Py][NTf_2]

M/g mol^{-1} = 418.33

ρ (298 K) / g cm^{-3} = 1.5618 [29]

solute	δ_1 [a]/MPa$^{0.5}$	Y [b]
hexane	14.9	0.0579
heptane	15.2	0.0621
octane	15.4	0.0655
nonane	15.7	0.069
decane	15.8	0.0706
methanol	29.4	0.3260
ethanol	26.1	0.2599

	solute	δ_1 [a]/MPa$^{0.5}$	Y [b]
	1-propanol	24.5	0.2276
	butanol	23.3	0.2058

[a] Calculated according to Equation (S12) using vaporisation enthalpies from compilation [26] and using experimental data from [28]

[b] The left part of Equation (S13): Y = $(\delta_1)^2/(RT) - \chi_{12}/V_1^*$

Table S7

Data used for regression with Equation (S13) for [N-C_3OH-Py][DCA]: solubility parameters δ_1 (at 298.15 K) of different solutes and the left part of Equation (S13).

[N-C_3OH-Py][DCA]	solute	δ_1 [a]/MPa$^{0.5}$	Y [b]
M/g mol^{-1} = 204.23	hexane	14.9	0.0452
ρ (298 K) / g cm^{-3} = 1.17101 [30]	heptane	15.2	0.0506
	octane	15.4	0.0550
	nonane	15.7	0.0594
	decane	15.8	0.0622
	methanol	29.4	0.3436

[a] Calculated according to Equation (S12) using vaporisation enthalpies from compilation [26] and using experimental data from [30].

[b] The left part of Equation (S13): Y = $(\delta_1)^2/(RT) - \chi_{12}/V_1^*$

Table S8

Results of regression with Equation (S13) for [N-C_3OH-Py][NTf_2] and [N-C_3OH-Py][DCA][a]

	[N-C_3OH-Py][NTf_2]	[N-C_3OH-Py][DCA]
slope	22.7	25.5
intercept	23.2	25.6
average	23.0	25.5
$\Delta_l^g H_m^o(T) = [\delta_2^2 \times V_m + RT]$	143.6	116.3
F [22]	1.00	1.41
$\Delta_l^g H_m^o(298.15\ K) = \Delta_l^g H_m^o(T) \times F$	143.6×1.0 =143.6±3.0	116.3×1.41 =163.9±3.0

[a] Enthalpies of vaporisation are given in kJ·mol^{-1}. Uncertainties are expressed as the twice standard deviation.

Table S9.

Compilation of Data Used for Evaluation of the Inter-Molecular Hydrogen Bonding in linear Amines, and Alkanes (at 298 K, in kJ·mol^{-1})[a]

Homomorph	$\Delta_l^g H_m^o$(exp)[26]	Homomorph	$\Delta_l^g H_m^o$(exp) [26]	HB_{inter} [b]
1-propanamine	31.3	n-butane	22.4	-8.9
1-butanamine	35.7	n-pentane	26.7	-9.0
1-pentanamine	40.5	n-hexane	31.7	-8.8
1-hexanamine	45.3	n-heptane	36.5	-8.8
1-heptanamine	50.1	n-octane	41.5	-8.6

[a] Uncertainties are ± 0.2 kJ·mol^{-1} (twice the standard deviation)

[b] Measure of the inter-molecular hydrogen bonding (difference between column 2 and 4)

Assessments of vaporization enthalpies of N-alkyl-pyridinium methanesulfonates

The experimental vaporization enthalpies of [1-(2-hydroxyethyl)pyridinium][OMs] and [1-(3-hydroxypropyl)pyridinium][OMs] were measured by QCM in this work (see Table 4). However, for the calculation of the hydrogen bond strength, the data of the ionic liquids N-alkyl-pyridinium [OMs] are needed as a reference. The latter values were evaluated using two entirely different algorithms, as shown in Figures S3 and S4.

The idea of the first algorithm is to derive the enthalpic contribution for unsubstituted [Py] fragment connected to the [NTf2] anion. Having established the numerical values for anions $\Delta_l^g H_m^o$([NTf2]) = 31.6 kJ·mol^{-1} and $\Delta_l^g H_m^o$([OMs]) = 46.6 kJ·mol^{-1} in our previous work [31], we exchanged anions for [Py] (see Figure S3 (left) and attached the necessary alkyl chains to it by using the GA-values from Table S10 (see Figure S3, right).

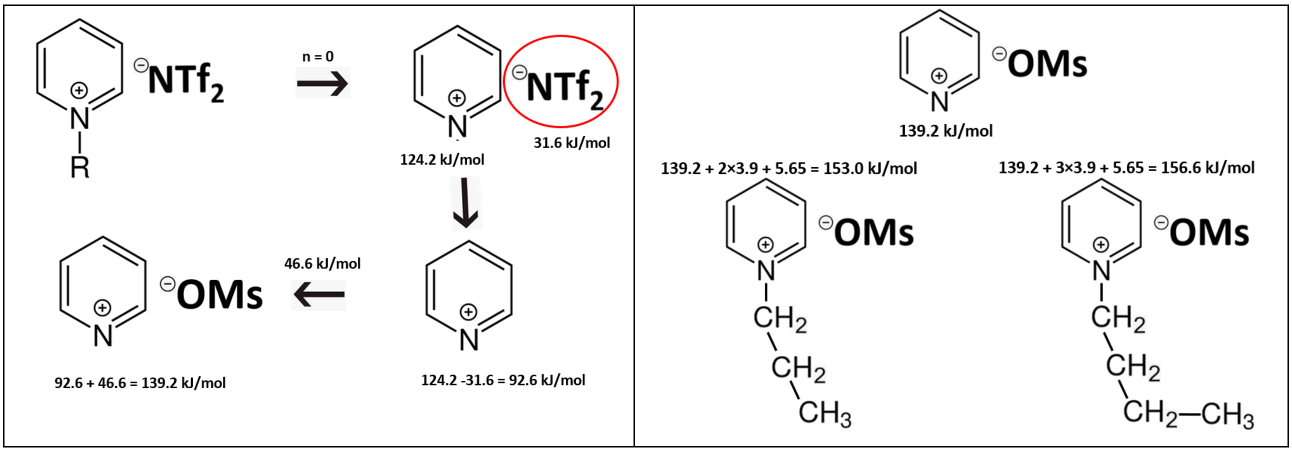

Figure S3 Algorithm for estimating the vaporization enthalpies, $\Delta_l^g H_m^o$(298.15 K), of N-propyl- and N-butyl pyridinium methanesulfonates starting from pyridinium based ILs [C_n-Py][NTf2]. Development of the [Py][OMs] fragment (left). Attachment of the alkyl chain to the [Py][OMs].

The enthalpic contribution for unsubstituted [Py] fragment connected to the [NTf2] anion was obtained from the linear correlation of $\Delta_l^g H_m^o$(298.15 K)-values with the number of carbon atoms in the alkyl chain within the homologue series of [C_n-Py][NTf2] series with the number of carbon atoms, *n*, in the alkyl chain attached to the cation nitrogen atom. The following correlation was obtained in Ref. [32]:

$$\Delta_l^g H_m^o(298.15\ \text{K})/\ \text{kJ·mol}^{-1} = 124.2 + 3.60\times n \quad (\text{ with } R^2 = 0.9958) \qquad \text{(S15)}$$

Apparently, the enthalpy contribution for the unsubstituted [Py] fragment linked to the [NTf2] anion was obtained by setting n = 0 (see Figure S3, left).

The idea of the second algorithm for estimating the vaporization enthalpies, $\Delta_l^g H_m^o$(298.15 K), of N-propyl- and N-butyl pyridinium methanesulfonates is to compare enthalpies of vaporisation of imidazolium based ILs [C_n-mim][NTf2] and pyridinium based ILs [C_n-Py][NTf2] with the comparable chain length (see Figure S4). Indeed, according to our experience [10], such a difference of 5.7 kJ·mol^{-1} remained constant and can be used to predict the unmeasured jet vaporisation enthalpies. As can be seen in Figures S3 and S4, the vaporisation enthalpies of N-propyl- and N-butyl pyridinium methanesulfonates derived by two independent routes agree well and they were averaged and applied in Table 4 (main text).

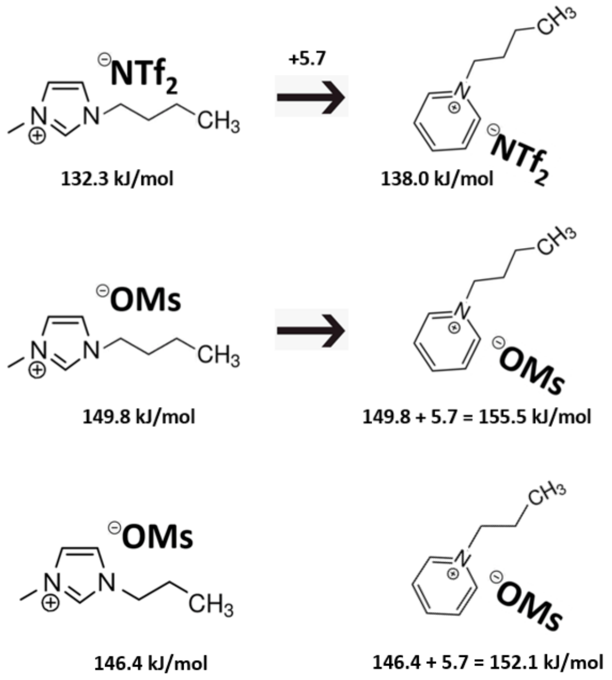

Figure S4 Algorithm for estimating the vaporization enthalpies, $\Delta_l^g H_m^o$(298.15 K), of N-propyl- and N-butyl pyridinium methanesulfonates starting from imidazolium based ILs [C_n-mim][NTf_2] (from. Ref. [33] and pyridinium based ILs [C_n-Py][NTf_2]. The experimental data for [C_n-mim][OMs] are from. Ref. [34].

As in Figures S3 and S4, the vaporization enthalpies of N-propyl and N-butylpyridinium methanesulfonates derived by two independent routes agree well and they were averaged and plotted in Table 4 (main text).

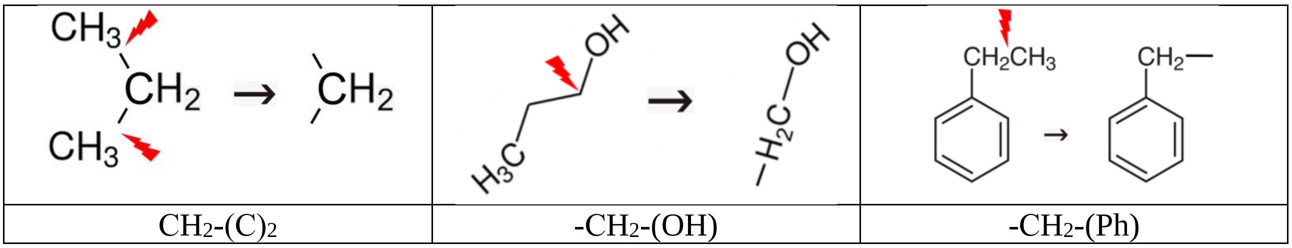

CH_2-$(C)_2$	-CH_2-(OH)	-CH_2-(Ph)

Figure S5 The development of groups for calculating the vaporisation enthalpies of hydroxy-alkyl-benzenes, hydroxy-alkyl-piperidines and hydroxy-alkyl-pyridinium [NTf_2] ionic liquids.

Table S10

Group-additivity values Γ_i for calculation of enthalpies of vaporization, $\Delta_l^g H_m^o$, at 298.15 K (in kJ mol^{-1}).

	$\Delta_l^g H_m^o$
	Γ_i [35]
**Alkanes**	
CH_3-(C)	5.65 [a]
CH_2-$(C)_2$	4.98
cyclic-CH_2-$(C)_2$	5.52 [b]
**Alcohols**	
HO-(CH_2)	31.8

CH_2-(OH)(C)	5.05 [c]
-CH_2-(OH)	36.85
**Amines**	
N-$(C)_3$	5.06
CH_2-(N_{tert})(C)	4.33 [d]
**[N-C_n-Py][NTf_2]**	
-CH_2-(Py-NTf_2)(C)	3.6 [32]
**“Centerpiece”**	
-CH_2-(Ph)	36.65
-CH_2-(Piperidine)	35.15
-CH_2-(Py-NTf_2)	125.25

[a] CH_3-(C) = CH_3-(N).

[b] Derived from enthalpy of vaporisation of cyclohexane, $\Delta_l^g H_m^o$ = 33.1±0.2 kJ mol^{-1} [26] divided by six.

[c] Derived from enthalpy of vaporisation of ethanol , $\Delta_l^g H_m^o$ = 42.5±0.2 kJ mol^{-1} [26] by subtracting the contributions HO-(CH_2) and CH_3-(C) from this table.

[d] Derived from enthalpy of vaporisation of tri-ethyl-amine , $\Delta_l^g H_m^o$ = 35.0±0.2 kJ mol^{-1} [26] by subtracting the contributions N-$(C)_3$ and CH_3-(C)×3 from this table, and finally divided by three.

References

1. Zaitsau, D.H.; Neumann, J.; Niemann, T.; Strate, A.; Paschek, D.; Verevkin, S.P.; Ludwig, R. Isolating the role of hydrogen bonding in hydroxyl-functionalized ionic liquids by means of vaporization enthalpies, infrared spectroscopy and molecular dynamics simulations. *Phys. Chem. Chem. Phys.* **2019**, *21*, 20308–20314, doi:10.1039/C9CP04337C.
2. Kulikov, D.; Verevkin, S.P.; Heintz, A. Determination of vapor pressures and vaporization enthalpies of the aliphatic branched C5 and C6 alcohols. *J. Chem. Eng. Data* **2001**, *46*, 1593–1600, doi:10.1021/je010187p.
3. Verevkin, S.P.; Emel´yanenko, V.N. Transpiration method: Vapor pressures and enthalpies of vaporization of some low-boiling esters. *Fluid Phase Equilib.* **2008**, *266*, 64–75, doi:10.1016/j.fluid.2008.02.001.
4. Verevkin, S.P.; Sazonova, A.Y.; Emel´yanenko, V.N.; Zaitsau, D.H.; Varfolomeev, M.A.; Solomonov, B.N.; Zherikova, K. V Thermochemistry of halogen-substituted methylbenzenes. *J. Chem. Eng. Data* **2015**, *60*, 89–103, doi:10.1021/je500784s.
5. Emel´yanenko, V.N.; Verevkin, S.P. Benchmark thermodynamic properties of 1,3-propanediol: Comprehensive experimental and theoretical study. *J. Chem. Thermodyn.* **2015**, *85*, 111–119, doi:10.1016/j.jct.2015.01.014.
6. Chickos, J.S.; Acree, W.E. Enthalpies of vaporization of organic and organometallic compounds, 1880-2002. *J. Phys. Chem. Ref. Data* **2003**, *32*, 519–878, doi:10.1063/1.1529214.
7. Chickos, J.S.; Hosseini, S.; Hesse, D.G.; Liebman, J.F. Heat capacity corrections to a standard state: a comparison of new and some literature methods for organic liquids and solids. *Struct. Chem.* **1993**, *4*, 271–278, doi:10.1007/BF00673701.
8. Scifinder, available online: https://scifinder.cas.org/.
9. Nichols, N.; Wadsö, I. Thermochemistry of solutions of biochemical model compounds 3. Some benzene derivatives in aqueous solution. *J. Chem. Thermodyn.* **1975**, *7*, 329–336, doi:10.1016/0021-9614(75)90169-X.
10. Zaitsau, D.H.; Yermalayeu, A.V.; Emel’yanenko, V.N.; Verevkin, S.P.; Welz-Biermann, U.; Schubert, T. Structure-property relationships in ILs: A study of the alkyl chain length dependence in vaporisation enthalpies of pyridinium based ionic liquids. *Sci. China Chem.* **2012**, *55*, 1525–1531, doi:10.1007/s11426-012-4662-2.
11. Diedrichs, A.; Gmehling, J. Measurement of heat capacities of ionic liquids by differential scanning calorimetry. *Fluid Phase Equilib.* **2006**, *244*, 68–77,

doi:http://dx.doi.org/10.1016/j.fluid.2006.03.015.
12. Ahmadi, A.; Haghbakhsh, R.; Raeissi, S.; Hemmati, V. A simple group contribution correlation for the prediction of ionic liquid heat capacities at different temperatures. *Fluid Phase Equilib.* **2015**, *403*, 95–103, doi:10.1016/J.FLUID.2015.06.009.
13. Zaitsau, D.H.; Yermalayeu, A.V.; Emel´yanenko, V.N.; Schulz, A.; Verevkin, S.P. Thermochemistry of pyridinium based ionic liquids with tetrafluoroborate anion. **2017**, *643*, 87–92, doi:10.1002/zaac.201600335.
14. Ribeiro da Silva, M.A.V.; Cabral, J.I.T.A. Standard molar enthalpies of formation of 1-methyl-2-piperidinemethanol, 1-piperidineethanol, and 2-piperidineethanol. *J. Chem. Thermodyn.* **2006**, *38*, 1461–1466, doi:10.1016/j.jct.2006.01.005.
15. Lepori, L.; Matteoli, E.; Gianni, P. Vapor pressure and its temperature dependence of 28 organic compounds: cyclic amines, cyclic ethers, and cyclic and open chain secondary alcohols. *J. Chem. Eng. Data* **2017**, *62*, 194–203, doi:10.1021/acs.jced.6b00576.
16. Verevkin, S.P. Thermochemistry of amines: Experimental standard molar enthalpies of formation of N-alkylated piperidines. *Struct. Chem.* **1998**, *9*, 113–119, doi:10.1023/A:1022459803871.
17. Zhuravleva, I.L. Evaluation of the polarity and boiling points of nitrogen-containing heterocyclic compounds by gas chromatography. *Russ. Chem. Bull.* **2000**, *49*, 325–328, doi:10.1007/BF02494682.
18. Verevkin, S.P.; Zaitsau, D.H.; Emel´yanenko, V.N.; Heintz, A.; A new method for the determination of vaporization enthalpies of ionic liquids at low temperatures. *J. Phys. Chem. B* **2011**, *115*, 12889–12895, doi:10.1021/jp207397v.
19. Sauerbrey, G. Verwendung von Schwingquarzen zur Wägung dünner Schichten und zur Mikrowägung. *Zeitschrift fur Phys.* **1959**, *155*, 206–222, doi:10.1007/BF01337937.
20. Zaitsau, D.H.; Yermalayeu, A..V.; Emel´yanenko, V.N.; Butler, S.; Schubert, T.; Verevkin, S.P. Thermodynamics of imidazolium-based ionic liquids containing PF6 anions. *J. Phys. Chem. B* **2016**, *120*, 7949–7957, doi:10.1021/acs.jpcb.6b06081.
21. Verevkin, S.P.; Ralys, R.V.; Zaitsau, D.H.; Emel´yanenko, V.N.; Schick, C. Express thermo-gravimetric method for the vaporization enthalpies appraisal for very low volatile molecular and ionic compounds. *Thermochim. Acta* **2012**, *538*, 55–62, doi:10.1016/j.tca.2012.03.018.
22. Verevkin, S.P. Imidazolium based ionic liquids: Unbiased recovering of vaporization enthalpies from infinite-dilution activity coefficients. *Molecules* **2021**, *26*, 5873, doi:10.3390/molecules26195873.
23. Flory, P.J. *Principles of polymer chemistry*; Cornell University Press,: Ithaca, N.Y., 1953;
24. Yoo, B.; Afzal, W.; Prausnitz, J.M. Solubility parameters for nine ionic liquids. *Ind. Eng. Chem. Res.* **2012**, *51*, 9913–9917, doi:10.1021/ie300588s.
25. Hansen, C.M. 50 Years with solubility parameters—past and future. *Prog. Org. Coatings* **2004**, *51*, 77–84, doi:10.1016/j.porgcoat.2004.05.004.
26. Majer, V.V.; Svoboda, V.V.; Kehiaian, H. v. *Enthalpies of vaporization of organic compounds: A critical review and data compilation*; Blackwell Scientific Publications: Oxford, 1985; ISBN 0632015292.
27. Lide, D.R. *CRC handbook of chemistry and physics : a ready-reference book of chemical and physical data*; 90th ed.; CRC Press, 2009; ISBN 9781420090840.
28. Marciniak, A. Activity coefficients at infinite dilution and physicochemical properties for organic solutes and water in the ionic liquid 1-(3-hydroxypropyl)pyridinium bis(trifluoromethylsulfonyl)-amide. *J. Chem. Thermodyn.* **2011**, *43*, 1446–1452, doi:10.1016/j.jct.2011.04.018.
29. Marciniak, A. The Hildebrand solubility parameters of ionic liquids — Part 2. *Int. J. Mol. Sci.* **2011**, *12*, 3553–3575, doi:10.3390/ijms12063553.
30. Domańska, U.; Wlazło, M.; Karpińska, M.; Zawadzki, M. Separation of binary mixtures hexane/hex-1-ene, cyclohexane/cyclohexene and ethylbenzene/styrene based on limiting activity coefficients. *J. Chem. Thermodyn.* **2017**, *110*, 227–236,

doi:10.1016/j.jct.2017.03.004.
31. Fumino, K.; Wulf, A.; Verevkin, S.P.; Heintz, A.; Ludwig, R. Estimating enthalpies of vaporization of imidazolium-based ionic liquids from far-infrared measurements. *ChemPhysChem* **2010**, *11*, 1623–1626, doi:10.1002/cphc.201000140.
32. Verevkin, S.P.; Zaitsau, D.H.; Ludwig, R. Aprotic ionic liquids: A framework for predicting vaporization thermodynamics. *Molecules* **2022**, *27*, 2321, doi:10.3390/molecules27072321.
33. Verevkin, S.P.; Zaitsau, D.H.; Emel´yanenko, V.; Yermalayeu, A.V.; Schick, C.; Liu, H.; Maginn, E.J.; Bulut, S.; Krossing, I.; Kalb, R.; et al. Making sense of enthalpy of vaporization trends for ionic liquids: new experimental and simulation data show a simple linear relationship and help reconcile previous data. *J. Phys. Chem. B* **2013**, *117*, 6473–6486, doi:10.1021/jp311429r.
34. Zaitsau, D.H.; Yermalayeu, A.V.; Pimerzin, A.A.; Verevkin, S.P. Imidazolium based ionic liquids containing methanesulfonate anion: comprehensive thermodynamic study. *Chem. Eng. Res. Desig* **2018**, *137*, 164–173, doi:10.1016/J.CHERD.2018.07.003.
35. Ducros, M.; Gruson, J.F.; Sannier, H.; Velasco, I. Estimation des enthalpies de vaporisation des composes organiques liquides. Partie 2. Applications aux ethersoxydes, thioalcanes, cetones et amines. *Thermochim. Acta* **1981**, *44*, 131–140, doi:10.1016/0040-6031(81)80035-4.
